# Evaluating the concordance between BICLA and SRI4 in patients with systemic lupus erythematosus from the placebo arms of the EXPLORER and ATHOS trials

**DOI:** 10.1136/lupus-2024-001483

**Published:** 2025-05-19

**Authors:** Anca D Askanase, Edward M Vital, Oliver Meier, Armando Turchetta, Huiyan (Ashley) Mao, Justine Maller, Jorge A Ross Terres, Maria Dall’Era

**Affiliations:** 1 Columbia University Irving Medical Center, New York University, New York City, New York, USA; 2 University of Leeds, Leeds Institute of Rheumatic and Musculoskeletal Medicine, Leeds, UK; 3 NIHR Leeds Biomedical Research Centre, Leeds Teaching Hospitals NHS Trust, Leeds, UK; 4 F. Hoffmann-La Roche Ltd, Basel, Switzerland; 5 Hoffmann-La Roche Ltd, Mississauga, Ontario, Canada; 6 Genentech, Inc, South San Francisco, California, USA; 7 University of California San Francisco, San Francisco, California, USA

**Keywords:** Lupus Erythematosus, Systemic, Outcome Assessment, Health Care, Arthritis

## Abstract

**ABSTRACT:**

**Objective:**

The British Isles Lupus Assessment Group (BILAG)-based Composite Lupus Assessment (BICLA) and the Systemic Lupus Erythematosus Responder Index 4 (SRI4) responses are the most common primary endpoints in SLE clinical trials. We examined the concordance and the reasons for discordance in BICLA/SRI4 responses in participants with SLE receiving placebo and standard of care (SOC) in two randomised controlled trials.

**Methods:**

This post-hoc analysis included data from the placebo arm (participants treated with SOC) of the EXPLORER (n=87; NCT00137969) and ATHOS (n=80; NCT02908100) trials. Disease activity was measured using BILAG and SELENA-SLEDAI (Safety of Estrogens in Lupus Erythematosus National Assessment-Systemic Lupus Erythematosus Disease Activity Index) in the EXPLORER trial and BILAG-2004 and SLEDAI-2000K in the ATHOS trial. For this analysis, participants were classified as responders or non-responders based on BICLA and SRI4 and the presence of intercurrent events. Concordance and discordance frequencies between BICLA and SRI4 were determined.

**Results:**

In EXPLORER, the BICLA response rates were lower than the SRI4 response rates (29.9% vs 41.4% at week 52, respectively), whereas in ATHOS the BICLA and SRI4 response rates were similar (41.2% vs 43.8% at week 48, respectively). The overall BICLA/SRI4 concordance (Cohen’s κ score) was moderate (0.46 in EXPLORER and 0.54 in ATHOS at weeks 52 and 48, respectively). At weeks 52 and 48, BICLA+/SRI4− and BICLA−/SRI4+ discordance, respectively, was 6.9% and 18.4% in EXPLORER and 10.0% and 12.5% in ATHOS. In an analysis of ATHOS subgroups based on the presence or absence of rash at baseline, BICLA response was higher at week 48 in participants with arthritis only than in those with arthritis and mucocutaneous comanifestations (63.2% vs 33.9%, respectively). BICLA−/SRI4+ and BICLA+/SRI4− discordance was lower in participants with low compared with normal complement at baseline.

**Conclusions:**

BICLA and SRI4 may be discordant in SLE trials. Arthritis and rash were the primary drivers of the discordance, and serology influenced BICLA and SRI4 response, suggesting the need for evaluation of multiple efficacy endpoints rather than a single measure.

**WHAT IS ALREADY KNOWN ON THIS TOPIC**

- There is a great deal of variability in how disease activity in patients with SLE is scored on the British Isles Lupus Assessment Group-based Composite Lupus Assessment (BICLA) and the Systemic Lupus Erythematosus Responder Index 4 (SRI4).
- The variability complicates the evaluation of efficacy and analysis of clinical trial data and can impact monitoring of disease progression for clinical studies.

**WHAT THIS STUDY ADDS**

- In this post-hoc analysis, participant-level data indicated that arthritis in isolation or coexistent with rash at baseline drives discordance between BICLA and SRI4 differently.
- Low complement levels are associated with low placebo response rates and increased concordance of BICLA and SRI4 responses.

**HOW THIS STUDY MIGHT AFFECT RESEARCH, PRACTICE OR POLICY**

- The results emphasise the need for a more comprehensive evaluation of efficacy in patients with SLE, rather than relying on a single measure.

## Introduction

 Due to the complexity and multiorgan involvement in the pathology of SLE, the efficacy of SLE therapies is often assessed using composite measures. The British Isles Lupus Assessment Group (BILAG)-based Composite Lupus Assessment (BICLA) and the Systemic Lupus Erythematosus Responder Index 4 (SRI4) responses are the most common outcome measures used in SLE randomised controlled trials (RCTs). However, there is a great deal of variability in how patients’ disease activity is scored on each of these scales, which complicates the evaluation of efficacy and analysis of clinical trial data and can impact monitoring of disease progression for clinical care.

There are key differences between SRI4 and BICLA. SRI4 is primarily based on improvement according to the Systemic Lupus Erythematosus Disease Activity Index (SLEDAI), in which each feature has a fixed number of points assigned to it, if deemed present (active due to SLE per the clinician assessor). For example, arthritis always scores 4 points (even if only of moderate severity), and resolution of arthritis is sufficient for a 4-point improvement per SRI4. In contrast, rash only scores 2 points (even if very severe), and resolution of this feature alone is insufficient for a 4-point improvement and classification as a responder per SRI4. Additionally, SLEDAI does not account for clinically meaningful partial improvement of active features (activity must completely resolve to no longer attribute points to a particular manifestation) and cannot capture worsening in the original active items or in new uncommon items. For this reason, SRI4 includes BILAG and physician Visual Analogue Scale to exclude worsening. There are points attributable for serological abnormalities only on SLEDAI—2 points for high anti-double-stranded DNA (dsDNA) antibody and 2 points for low complement—so that improvement of both can qualify for SRI4 even in the absence of clinical improvement.

BICLA, on the other hand, is primarily based on improvement in active disease severity according to the BILAG Index, in which each organ domain is graded A–E, with category A being the most severe and category E being never involved. To be considered a BICLA responder, all active features must improve by at least one letter grade (eg, all A category domains must improve to at least a B category), and clinically meaningful but partial improvement still contributes to a BICLA response. The immunological abnormalities included in SLEDAI are not captured at all using BILAG. BICLA also checks SLEDAI and physician Visual Analogue Scale for worsening; however, unlike SRI4, BICLA includes a therapy term for exclusion of worsening, that is, patients with improvement in BILAG but with increase in glucocorticoid doses above the protocol rules are not considered BICLA responders.

Kim *et al* showed that patients are less likely to achieve a response at week 52 using BICLA than SRI4, with response rates ~8% lower (38.0% vs 45.9%, respectively).^1^ In a post-hoc analysis of the placebo arms of six SLE RCTs, BICLA and SRI4 concordance varied by clinical trial (Cohen’s kappa (κ) range, 0.39–0.76).^2^ Overall, the agreement between BICLA and SRI4 was higher at week 52 than at week 24. Discordance between BICLA and SRI4 responses across the trials ranged from 11.8% to 30.3%, and there were more SRI4 responders than BICLA responders. The non-response to BICLA and SRI4 was largely due to a lack of clinical improvement on the BILAG Index or SLEDAI, respectively, as well as study withdrawal or concomitant medication violations. Analysis of patient-level data could improve understanding of the reasons for the discrepancy between BICLA and SRI4 responses. Additionally, the reasons underlying BICLA and SRI4 discordance may be elucidated by investigating the influence of organ domain involvement and serological factors.

In this post-hoc analysis, which included separate analyses of the EXPLORER and ATHOS trials, we estimated concordance in BICLA and SRI4 outcome measures in participants with SLE receiving placebo and standard of care (SOC).^3 4^ Because our aim was to compare efficacy evaluations within a population of patients, the specific investigational therapies, SOC therapies or effect sizes between treatment arms were not important to our analysis; only the existence of a range of responses to any kind of therapy was considered. We also investigated the underlying reasons for discordance by examining the influence of organ domains and serological factors at the participant level.

## Methods

### Patient population and RCT study designs

Data were analysed from participants with evaluable BICLA and SRI4 responses who were receiving SOC and enrolled in the placebo arms of the EXPLORER (n=87; NCT00137969) and ATHOS (n=80; NCT02908100) RCTs. Patient baseline characteristics and entry criteria for the RCTs have been described previously.^3 4^ Both were global studies that included both US and ex-US patient populations. The ATHOS study recruited 57 people with SLE (22%) from the USA and Western Europe (UK, Spain, France, Portugal and Germany) and 202 people with SLE (78%) from the rest of the world (ROW). In the ATHOS placebo arm, there were 19 (22.6%) and 65 (77.4%) patients with SLE from the USA and ROW, respectively.

In EXPLORER, active disease at screening was defined by ≥1 domain with a BILAG score of A or ≥2 domains with a BILAG score of B. In ATHOS, active disease was defined as a SLEDAI-2000K (SLEDAI-2K) score of ≥8 at screening only, with a clinical SLEDAI-2K score of ≥4.0 at both screening and day 1.

The trials applied two different glucocorticoid taper protocols. EXPLORER had an oral glucocorticoid target dose of prednisone (or equivalent) ≤10 mg/day by week 12 and ≤5 mg/day by week 52. ATHOS had an oral glucocorticoid target of <10 mg/day at both weeks 12 and 36, followed by a 12-week stability window after each target time point. Disease activity was measured using BILAG and the Safety of Estrogens in Lupus Erythematosus National Assessment-Systemic Lupus Erythematosus Disease Activity Index (SELENA-SLEDAI) in EXPLORER and BILAG-2004 and SLEDAI-2K in ATHOS.

### Data analysis

BICLA and SRI4 data from week 52 in EXPLORER and week 48 in ATHOS were analysed separately. Participants were classified as either responders or non-responders based on the BICLA and SRI4 composite indices and the presence of intercurrent events. In the ATHOS population, participants were imputed as non-responders based on the protocol-defined criteria for early study discontinuation and rescue therapy. In the EXPLORER population, participants who discontinued early for any reason were considered non-responders as per protocol; rescue medication was not considered. BICLA required an improvement in all BILAG domains rated A or B at baseline, and SRI4 required an improvement of ≥4 points on SLEDAI. Both BICLA and SRI4 responses required that no new activity occurred in previously uninvolved or improved BILAG domains (ie, no new BILAG A involvement or no more than one new BILAG B manifestation), no net increase in SLEDAI-2K and no worsening of Physician Global Assessment by ≥0.3 points. Any remaining missing data were imputed via last observation carried forward at the item level of BICLA and SRI4 composite endpoints. Concordance and discordance frequencies between BICLA and SRI4 were determined. Overall concordance between BICLA and SRI4 was estimated using Cohen’s κ score.

ATHOS participant-level data from the tender and swollen joint count (TSJC) and the Cutaneous Lupus Disease Area and Severity Index (CLASI) were also examined to better understand domain-specific reasons for discordance in the composite outcome measures. Immunoassays were used to analyse C3, C4 and CH50 (Siemens and Covance). The cut-off for positivity was defined as above the lower limit of normal for the testing laboratory. Immunoassays were used to analyse anti-dsDNA antibodies (Inova Diagnostics and Covance). The cut-off for positivity for anti-dsDNA antibody was defined as >25% by Farr assay or above the normal range for the testing laboratory.

### Patient and public involvement

Patients and the public were not directly involved in the design of this study or in the analysis of data.

## Results

### BICLA and SRI4 response rates and concordance in EXPLORER and ATHOS

**Figure 1:**
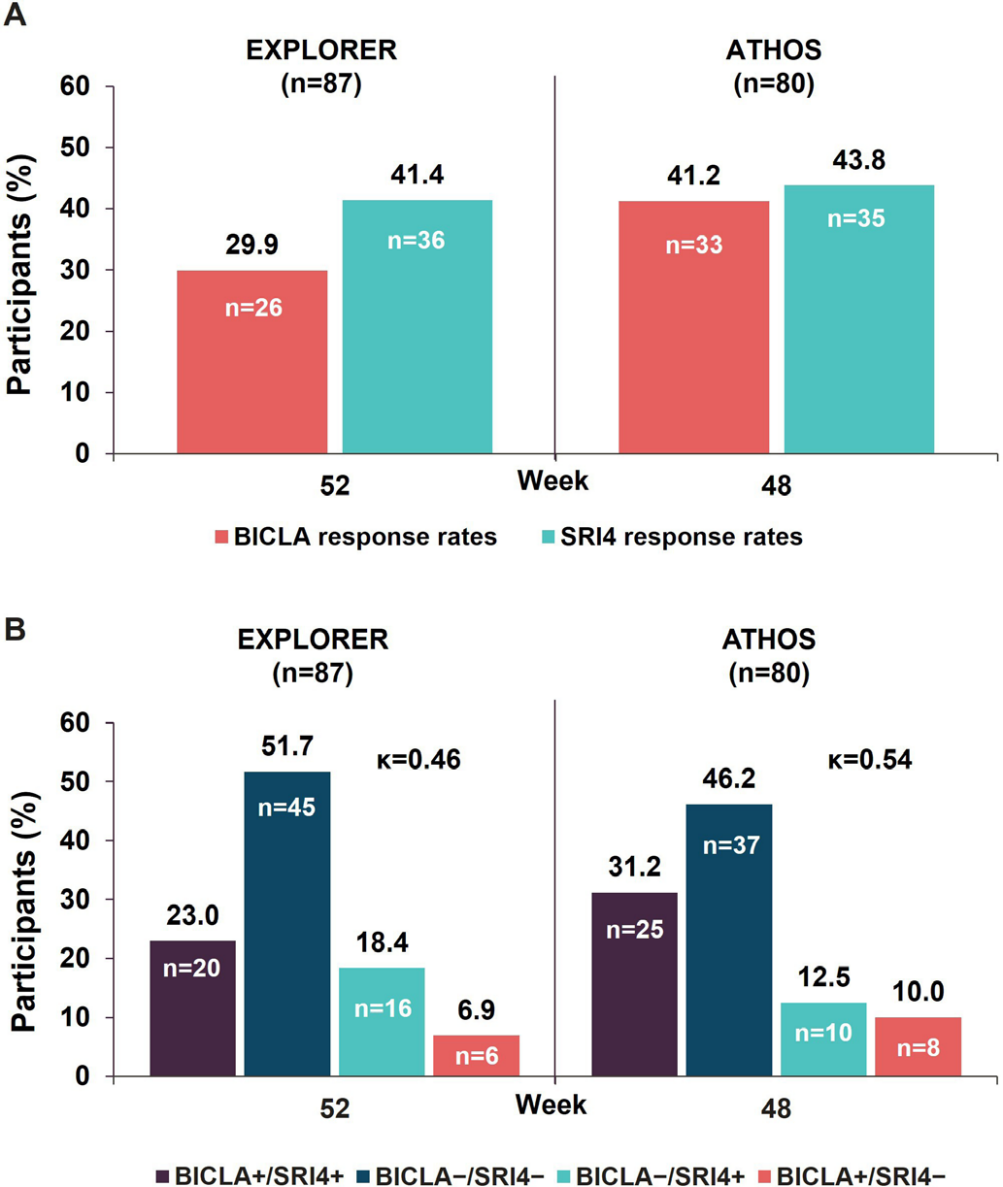
(**A**) SRI4 and BICLA response rates and (**B**) BICLA and SRI4 concordance and discordance in the placebo arms of the EXPLORER and ATHOS trials. ‘+’ indicates responder and ‘−’ indicates non-responder based on the BICLA and SRI4 composite indices. BICLA, British Isles Lupus Assessment Group-based Composite Lupus Assessment; SRI4, Systemic Lupus Erythematosus Responder Index 4.

A total of 87 participants from the placebo arm of the EXPLORER trial and 80 participants from the placebo arm of the ATHOS trial had evaluable BICLA and SRI4 responses and were included in the analysis. In the placebo arm of EXPLORER, the BICLA response rates were lower than the SRI4 response rates (29.9% vs 41.4%) at week 52. In the placebo arm of ATHOS, the BICLA and SRI4 rates were similar (41.2% vs 43.8%) at week 48 (figure 1A). The overall BICLA and SRI4 concordance (Cohen’s κ score) was moderate, with 0.46 in EXPLORER and 0.54 in ATHOS at weeks 52 and 48, respectively. The concordance was primarily driven by the participants whose disease activity did not improve on either SLEDAI or BILAG instruments (figure 1B).

In EXPLORER, BICLA responder (BICLA+)/SRI4 non-responder (SRI4–) discordance was 6.9% and BICLA non-responder (BICLA−)/SRI4 responder (SRI4+) discordance was high at 18.4%, which corresponds with the lower BICLA response rate of 29.9% vs 41.4% for SRI4. Compared with the BICLA−/SRI− concordant subgroup (51.7%), the percentage of participants who responded by both BICLA and SRI4 was low at 23.0% (figure 1B). However, in ATHOS, the lower BICLA+/SRI4− (10.0%) and BICLA−/SRI4+ (12.5%) discordance was in line with the similar BICLA and SRI4 response rates at week 48 (41.2% and 43.8%, respectively) (figure 1A,B). The number of participants in the concordant subgroups, that is, BICLA+/SRI4+ and BICLA–/SRI4–, was much higher than in the discordant subgroups (figure 1B).

### Discordance in ATHOS by investigating arthritis and rash domains

To better elucidate the influence of the organ domains on BICLA/SRI4 discordance, we investigated patient-level data for the TSJC and the CLASI activity score in ATHOS.^5 6^ These analyses were not performed for the EXPLORER study, as such patient-level data were not available.

#### BICLA+/SRI4− discordance

**Figure 2:**
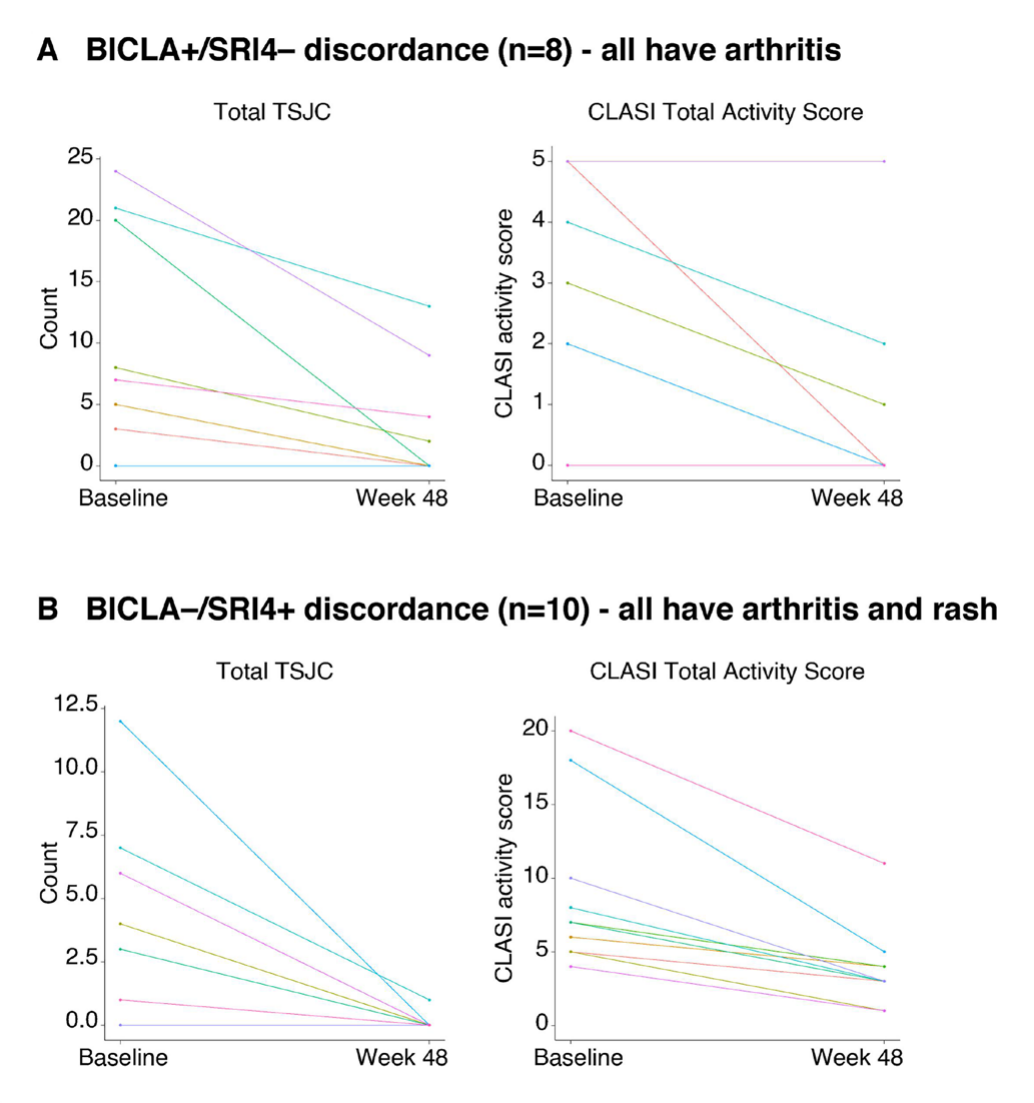
Total TSJC and CLASI activity scores in (**A**) BICLA+/SRI4− discordant participants and (**B**) BICLA−/SRI4+ discordant participants. ‘+’ indicates responder and ‘−’ indicates non-responder based on the BICLA and SRI4 composite indices. BICLA, British Isles Lupus Assessment Group-based Composite Lupus Assessment; CLASI, Cutaneous Lupus Disease Area and Severity Index; SRI4, Systemic Lupus Erythematosus Responder Index 4; TSJC, tender and swollen joint count.

Participants with BICLA+/SRI4– discordance showed a gradual reduction in the total number of active joints (defined as both swollen and tender per the TSJC form) from baseline; however, because active arthritis persisted in two or more joints at week 48, they retained the 4-point attribution for arthritis, as defined by SLEDAI, and thus did not meet the definition for an SRI4 response (figure 2A). Interestingly, half of the participants had coexisting signs of inflammation other than swelling, namely effusion, warmth or erythema. If only swelling was used as a sign of inflammation, half of the BICLA+/SRI4– group would have achieved an SRI4 response and the discordance would have been reduced by half (figure 2A). Although four participants had an active joint count of 0 per the TSJC form, arthritis was still scored as present on SLEDAI due to coexisting signs of inflammation other than swelling. In addition, half of the participants had active mucocutaneous manifestations per CLASI that improved or resolved. On SLEDAI, the resolution of one of the three mucocutaneous items (rash, alopecia or mucosal ulcers) translates to a 2-point reduction. One participant (purple line) had improvements in rash but worsening alopecia, corresponding to 5 points at both time points on the CLASI activity score. This was also represented by a BILAG mucocutaneous letter change from B to C (reflecting improvement in active disease severity). Another participant (red line) had alopecia and rash at baseline, which resolved by week 48, but developed low complement levels (a new 2-point attribution on SLEDAI), which prevented a 4-point improvement in the total SLEDAI score.

**Figure 3:**
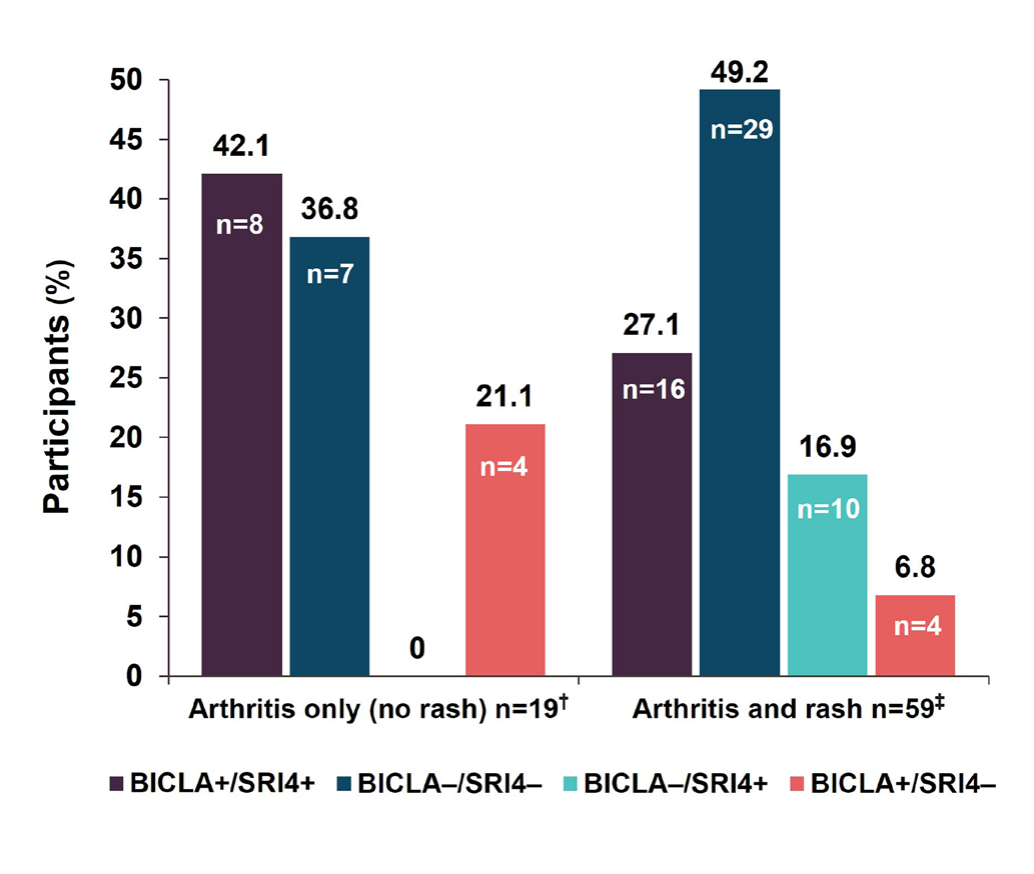
Discordance in two mutually exclusive subgroups with arthritis based on the absence or presence of rash at baseline in the ATHOS study. A total of 78 out of 80 participants had arthritis manifestations on SLEDAI at baseline and BILAG A or B musculoskeletal manifestations (two participants had isolated mucocutaneous manifestations without arthritis). ‘+’ indicates responder and ‘−’ indicates non-responder based on the BICLA and SRI4 composite indices. †Arthritis (SLEDAI) without BILAG A or B mucocutaneous at baseline (may have alopecia or mucosal ulcers). ‡Arthritis (SLEDAI) with BILAG A or B mucocutaneous at baseline. BICLA, British Isles Lupus Assessment Group-based Composite Lupus Assessment; BILAG, British Isles Lupus Assessment Group; SLEDAI, Systemic Lupus Erythematosus Disease Activity Index; SRI4, Systemic Lupus Erythematosus Responder Index 4.

CLASI activity scores of 2 or 0 at baseline were not sufficient to register on SLEDAI. Some participants had identical trajectories for active joint counts and CLASI activity scores. In summary, if musculoskeletal and mucocutaneous manifestations improved partially and did not resolve, no point improvements were captured on SLEDAI. Thus, the 4-point SLEDAI improvement required for an SRI4 response was not achieved. Conversely, if all active organ domains improved partially, there was a letter-grade change on BILAG, indicative of a BICLA response (mucocutaneous: four participants changed from B to C, three changed from C to D and one had a stable E score; musculoskeletal: six participants changed from B to C and two from A to C). BICLA+/SRI4− discordance doubled from 10.0% of all participants at week 48 (figure 1B) to 21.1% of participants with stable arthritis based on SLEDAI (arthritis-only subgroup) (figure 3). BICLA+/SRI4− discordance (6.8%) was lower for participants with severe mucocutaneous involvement comanifesting with arthritis (arthritis and rash subgroup), since they would be more likely to achieve improvement per the BILAG Index (figure 3).

#### BICLA−/SRI4+ discordance

All participants with BICLA–/SRI4+ discordance showed complete resolution of arthritis to less than two joints by week 48. All had mucocutaneous comanifestations that were captured by SLEDAI. Either alopecia or mucosal ulcers resolved but not rash. The CLASI activity scores were generally high and did not improve sufficiently to achieve a letter-grade improvement on BILAG. All 10 participants had stable BILAG scores of B for mucocutaneous manifestations (figure 2B). In summary, isolated resolution of arthritis with persistent mucocutaneous activity was sufficient to achieve an SRI4 response. Conversely, since these participants did not show improvements across all active organ domains on BILAG, they did not achieve a BICLA response (figure 2B). In participants with arthritis only, BICLA−/SRI4+ discordance was not observed (0%) (figure 3). BICLA−/SRI4+ discordance was higher in participants with improving arthritis and comanifestations of stable rash (arthritis and rash subgroup; 16.9%) than in all participants (12.5%) at week 48 (figures1  3).

#### BICLA+/SRI4+ and BICLA−/SRI4− concordance

At week 48, a higher concordant BICLA+/SRI4+ response rate was observed in participants with isolated arthritis manifestations compared with the response rate of participants with severe mucocutaneous comanifestations (BILAG score of A or B) at baseline (42.1% vs 27.1%; figure 3). BICLA−/SRI4− concordant non-response was higher in participants with arthritis and rash comanifestations compared with those with arthritis only (49.2% vs 36.8%; figure 3).

#### All BICLA responders/non-responders or SRI4 responders/non-responders

**Figure 4:**
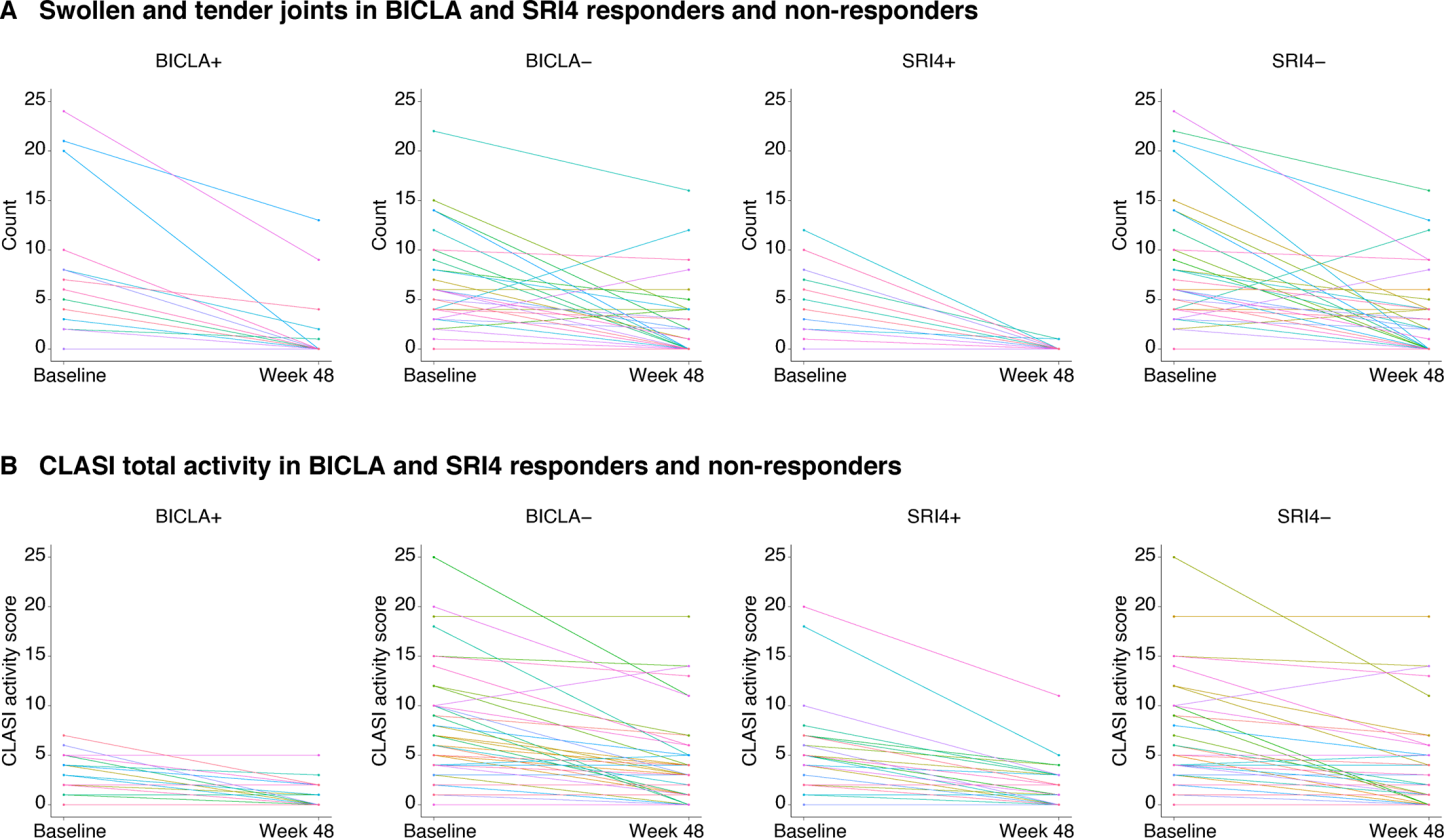
BICLA and SRI4 responder and non-responder scores in (**A**) TSJC and (**B**) CLASI activity. ‘+’ indicates responder and ‘−’ indicates non-responder based on BICLA and SRI4 composite indices. BICLA, British Isles Lupus Assessment Group-based Composite Lupus Assessment; CLASI, Cutaneous Lupus Disease Area and Severity Index; SRI4, Systemic Lupus Erythematosus Responder Index 4.

Examination of data from all participants, independent of the concordance/discordance groups, showed that BICLA responders had clear improvements in active joint counts and in CLASI activity scores. The baseline and week 48 TSJC and CLASI activity scores of BICLA responders were much lower than those of BICLA non-responders, which was more evident in the CLASI activity score (figure 4A,B). The findings for BICLA non-responders were more complex for participants with active joint counts and CLASI activity scores.

An SRI4 response was less clearly associated with CLASI activity scores at baseline. Although the CLASI activity scores improved gradually or remained stable, all SRI4 responders achieved resolution of arthritis (0–1 active joints) per the TSJC form (figure 4A,B). Baseline active joint counts were lower for SRI4 responders compared with non-responders. These findings suggest that an SRI4 response is more probable with lower active joints at baseline. Active joint counts and CLASI activity scores for SRI4 non-responders showed no clear pattern (figure 4A,B).

### Influence of serological factors on outcome–response concordance in ATHOS

**Figure 5:**
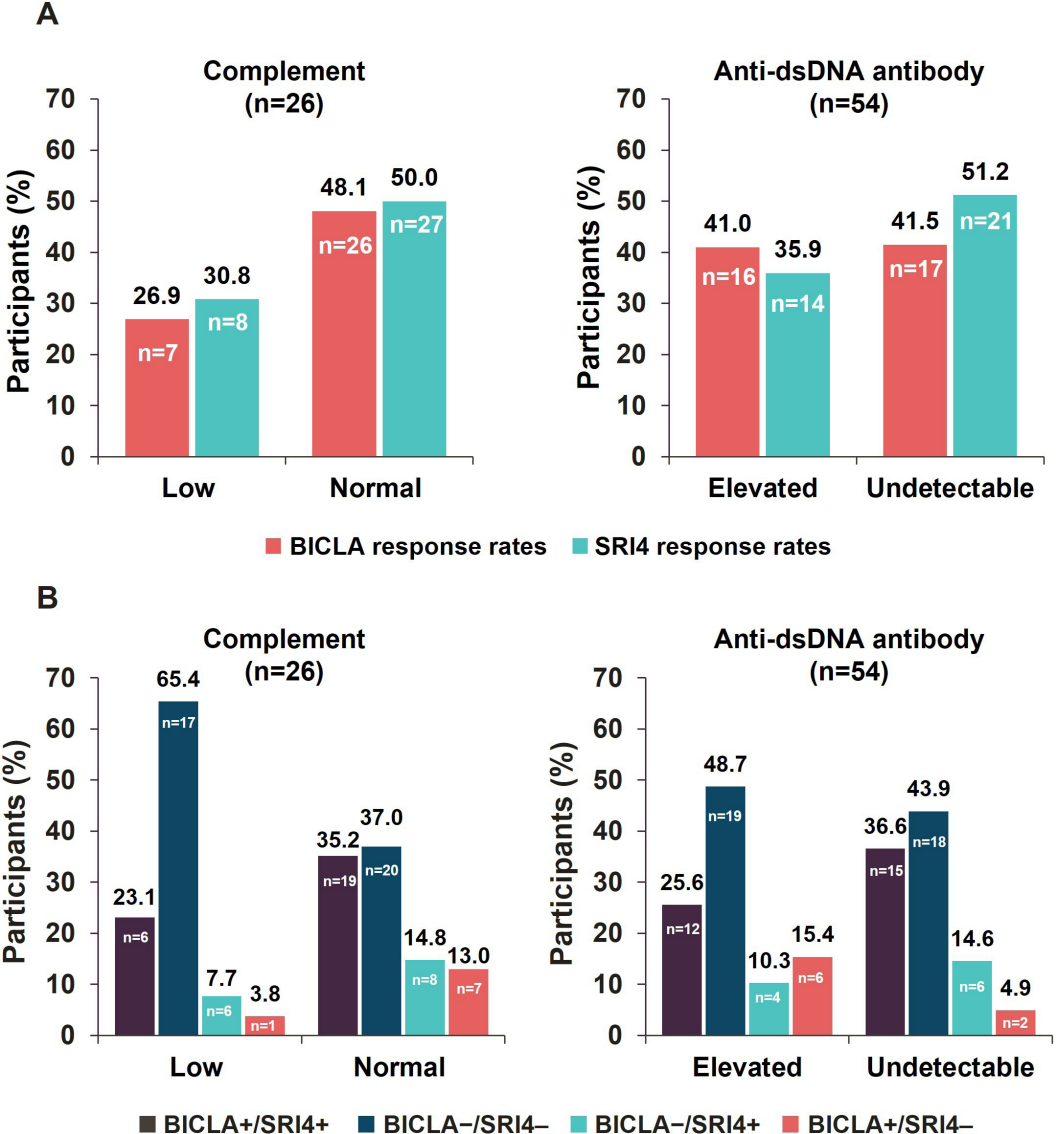
BICLA and SRI4 (**A**) response rates and (**B**) concordance based on baseline complement and anti-dsDNA antibody levels. ‘+’ indicates responder and ‘−’ indicates non-responder based on BICLA and SRI4 composite indices. BICLA, British Isles Lupus Assessment Group-based Composite Lupus Assessment; dsDNA, double-stranded DNA; SRI4, Systemic Lupus Erythematosus Responder Index 4.

Both BICLA and SRI4 response rates at week 48 were lower in participants with low complement levels at baseline than in participants with normal complement levels (26.9% vs 48.1% and 30.8% vs 50.0%, respectively) (figure 5A). Autoantibody positivity at baseline seemed to have less of an impact on BICLA response rates at week 48, which were similar between participants with undetectable (41.5%) and elevated (41.0%) anti-dsDNA antibody levels (figure 5A). Participants with undetectable anti-dsDNA antibody levels at baseline (and thus no 2-point attribution on SLEDAI) were more likely to achieve an SRI4 response at week 48 than participants with elevated anti-dsDNA antibody levels at baseline (51.2% vs 35.9%) (figure 5A). Participants with low complement at baseline achieved concordant BICLA+/SRI4+ responses less frequently than those with normal complement (23.1% vs 35.2%) and also demonstrated a much higher rate of non-response concordance (65.4% vs 37.0%) (figure 5B). Participants with high baseline anti-dsDNA antibody levels also achieved concordant responses less frequently compared with participants with undetectable levels at baseline (25.6% vs 36.6%); non-response concordance was only slightly higher (48.7% vs 43.9%) (figure 5B). BICLA−/SRI4+ and BICLA+/SRI4− discordance was lower in participants with low complement at baseline compared with participants with normal complement levels (7.7% vs 14.6% and 3.8% vs 13.0%, respectively) (figure 5B). Anti-dsDNA antibody positivity did not translate to lower discordance in either of the non-responder groups (figure 5B).

## Discussion

In this post-hoc analysis of participants from the placebo arms of two SLE trials, we found that BICLA/SRI4 concordance was higher in the ATHOS trial compared with the EXPLORER trial. Given the age of the EXPLORER study, which used an older version of the BILAG Index to trend disease activity and did not implement joint count or CLASI assessments, much of our focus was dedicated to the data obtained from the more recent ATHOS study. Our investigation of participant-level data revealed that lacking changes in arthritis or rash were the primary drivers of BICLA/SRI4 discordance, and the presence of low complement at baseline was associated with the highest rate of concordant non-response. The overall BICLA/SRI4 concordance for EXPLORER (0.46) and ATHOS (0.54) was in line with previously reported BICLA/SRI4 concordance across six RCTs, which showed variation by trial ranging from 0.39 to 0.76.^2^

The differences in BICLA/SRI4 concordance across clinical trials could in part be explained by differences in trial design, including versions of disease activity indices used. The newer versions of the BILAG and SLEDAI disease activity indices may have led to higher BICLA/SRI4 concordance in ATHOS relative to EXPLORER. Compared with the original BILAG, BILAG-2004 distributed the vasculitis items into the mucocutaneous and other domains, added ophthalmic and gastrointestinal sections and included numerical scoring and updates to haematology items.^7^,^9^ The original SLEDAI was modified to create the SELENA-SLEDAI to ensure that the descriptors capture changes in disease activity (and the inclusion of the SELENA-SLEDAI Flare Index). The SLEDAI-2K used in ATHOS was modified to allow for the documentation and scoring of ‘present’ objective, persistent active disease in domains that were previously scored only if they were based on new or recurrent events, and to increase usability in clinical trials. The definitions of arthritis, pleurisy, pericarditis, rash, alopecia, mucosal ulcers and proteinuria of >0.5 g/24 hours were revised.^9^,^12^

There were also differences in the glucocorticoid taper protocols between the EXPLORER and ATHOS studies, which could affect BICLA and SRI4 response rates; however, this would be unlikely to impact the probability of concordance between these two outcome measures. The significant BICLA/SRI4 discordance observed in ATHOS likely originates from the diverging nature of the instruments (gradual/partial improvement vs categorical improvement). In a previous study, comparison of BICLA and SRI4 against a physician’s global rating of clinically significant improvement suggested that BICLA was less sensitive in detecting improvements when multiple organs were involved at baseline, but could be more effective in assessing overall improvements in disease activity.^13^

More specific details of the organ systems involved could explain BICLA/SRI4 discordance, such as whether participants have arthritis only or have comanifestations of mucocutaneous rash.^13^ In participants with multiple active BILAG domains, all domains must improve at least partially to achieve a BICLA response, for example from A to B or from B to C.^7^,^9^ Conversely, achieving an SRI4 response requires only one important organ domain to improve by 4 points, which means a response can be achieved by improvement in arthritis alone, although the more binary nature of the scale requires a more complete improvement. However, some residual activity may still be present despite the organ item being rated absent (ie, arthritis, thrombocytopaenia, leucopenia not meeting the item definition). Further, participants with improvement in their joints but with significant persistent disease in their skin could achieve SRI4 but not BICLA. This may explain why there are more BICLA non-responders in the arthritis and rash subgroup than SRI4 non-responders. Scoring arthritis as present on SLEDAI because of features like tenderness or warmth, but without swelling, was recently shown to overscore disease activity compared with imaging-based confirmation of synovitis, which would not occur for the BILAG A and B scores.^14^

In the present study, evaluating participant-level data from the ATHOS study enabled a more detailed comparison of the disease activity indices and elucidated specific reasons underlying discordance in the response measures. SRI4 responders showed stability or improvement in their CLASI activity scores and resolution in all TSJCs to less than two joints. In contrast, although SRI4 non-responders had stability or improvement in their CLASI activity scores, there was no resolution of their active joint counts. Baseline active joint counts were lower for participants who achieved SRI4 responses compared with the SRI4 non-responders, suggesting that an SRI4 response is more likely to be achieved with standard therapy (ie, in the placebo arm) when participants have relatively few active joints at baseline.

BICLA responders showed improvements in both CLASI activity scores and active joint counts. Participants with partial improvements in CLASI and TSJC more frequently achieved BICLA than SRI4 responses. Baseline CLASI activity scores were much lower at baseline in BICLA responders compared with non-responders, suggesting that a BICLA response is more likely to be achieved in patients who enter a study with lower CLASI activity scores. The findings for BICLA non-responders are more complex. Participants with improvement in their arthritis but with persistent significant mucocutaneous disease activity achieved SRI4 but not BICLA, likely reflecting the more binary nature of SLEDAI and its impact on the SRI4 outcome.

SLEDAI counts low complement and elevated anti-dsDNA antibody levels towards the total score, whereas BILAG does not include these parameters. Improvement in these immunological components can thus lead to an SRI4 response but has no impact on BICLA response. While improvements in immunological activity without change in clinical activity are of debatable significance to patients and their providers, in the context of clinical trials, our findings suggest that active serology had an influence on BICLA/SRI4 concordance. The concordant response in ATHOS at week 48 (BICLA+/SRI4+) was lower in participants with either elevated anti-dsDNA antibody levels or low complement at baseline. This supports the proposed novel stringent endpoint of ‘dual BICLA and SRI4 response’.^15 16^ Discordance in participants with elevated anti-dsDNA antibody levels (10.3% and 15.4%, totalling 25.7%) was greater than in participants with low complement (7.7% and 3.8%, totalling 11.5%) at baseline. This finding is driven by the fact that significantly lower BICLA and SRI4 response rates were observed in participants with low complement at baseline, whereas participants with elevated anti-dsDNA antibody levels only showed lower SRI4 response. Thus, low baseline complement levels appear to be the more powerful serological surrogate for disease activity, which can lead to lower BICLA response rates despite this parameter not being included in BILAG. Participants with low complement were more likely to have higher disease activity in joints or skin at baseline, which prevented more patients from achieving SRI4 or BICLA response, respectively, as shown in the participant-level analysis. A post-hoc analysis investigating SLE treatment efficacy in patients with a SELENA-SLEDAI score ≥10 as well as low complement and/or positive anti-dsDNA antibodies suggested that only one of these serology biomarkers needed to be present to predict a positive treatment response.^17^ With a lower baseline SLEDAI score of ≥8 in the ATHOS study, low complement becomes the better predictor of concordant non-response in placebo-treated patients.

Due to differences in study design and the clinical measurements used, it was not possible to directly pool and compare the data from the ATHOS and EXPLORER studies in the present analysis. The underlying causes of discordance between BICLA and SRI4 are influenced by the organ domains involved, serological factors and the overall level of disease activity. In general, it is harder to achieve a response using BICLA, whereas SRI4 is more easily achieved. These endpoints agree most of the time, but they may disagree for certain subgroups of patients and levels of response, which may have different effects on the outcome depending on the trial population. A combined endpoint using both BICLA and SRI4 may reduce these uncertainties for future SLE trial designs, enabling trials to be conducted with fewer participants.^16^ This study looked at responses to standard therapy only, which may represent the natural course of this chronic, heterogeneous disease rather than the effects of the more targeted medications currently in development. When active treatments are used, these may favour improvements in certain organs, which may again influence discordance between endpoints.

SLE is a complex disease with many features that have different pathogenic mechanisms and are difficult to fully summarise into a single score. In several previous SLE trials, overall conclusions about efficacy have required evaluation of many endpoints, not just the primary endpoint. Although multiple instruments and endpoints are collected, hierarchical testing gives greater priority to some. These results emphasise the value of a comprehensive evaluation of efficacy data rather than relying on a single measure.

## Data Availability

Data are available upon reasonable request.
